# Directive emission from polymeric fluorophore with epsilon-near-zero squaraine molecular film

**DOI:** 10.1515/nanoph-2022-0763

**Published:** 2023-04-25

**Authors:** Kyu-Ri Choi, Minjae Kim, Jeong Weon Wu, Anthony D’Aléo, Yeon Ui Lee

**Affiliations:** Department of Physics, Chungbuk National University, Cheongju, Chungbuk, 28644, South Korea; Department of Physics, Ewha Womans University, Seoul, 03760, South Korea; Université de Strasbourg, CNRS, Institut de Physique et Chimie des Matériaux de Strasbourg, UMR 7504, F-67000 Strasbourg, France

**Keywords:** directive emission, epsilon-near-zero, metamaterials, organic plasmonics, photoluminescence

## Abstract

Enhanced directionality of photoluminescence emission has attracted attention due to its diverse application areas ranging from single-photon sources to fluorescence sensing and bio-imaging. Utilization of null phase advance in epsilon-near-zero (ENZ) medium is an important scheme to achieve the directive emission. Despite various designs proposed for ENZ-based directive emission, most of the ENZ mediums are restricted to subwavelength structures of metallic plasmonics or inorganic dielectrics. Here, we introduce an organic ENZ film placed on top of a polymeric fluorophore film to demonstrate a directive emission. By taking advantage of the structural coherence in the P3HT film and the ENZ response in the squaraine molecular film, 42 % increase in directive emission is achieved. Capability to control directive emission with organic ENZ films is highly useful in applications requiring bio-compatibility of a fluorophore-embedding medium.

## 1 Introduction

Directive emission of fluorophores is very useful in single-photon sources [1, 2], lab-on-a-chip devices [3], fluorescence sensing and imaging in photonics and biology [4, 5]. Therefore, tremendous efforts have been made in the last two decades of research to enhance the directionality of emissions. Schemes to achieve directive emission include a dipole antenna embedded in a dielectric slab Fabry–Perot cavity [6], construction of subwavelength metamaterial-based cavities [7], and placement of a monopole radiation source in a photonic crystal [8].

In an ENZ medium, the temporal and spatial variations of an electromagnetic (EM) wave decouples and the spatial distribution of time-varying electric field is uniform, corresponding to a null phase advance and a stretched wavelength. This leads to an enhanced spatial coherence of an EM wave inside an ENZ medium, which results in a wavefront of out-going EM waves from the ENZ slab following the flat surface of the ENZ slab, giving rise to a directive emission [9–11]. In other words, the utilization of the ENZ dispersion is an important scheme in achieving a directive emission. A variety of ENZ media has been introduced with corresponding ENZ spectral positions to demonstrate directive emission. Examples include ENZ metamaterials composed of copper grids at the microwave of *f* = 14.65 GHz [12], all-dielectric zero-index optical metamaterials consisting of stacked silicon-rods at *λ* ≈ 1,400 nm [13], and on-chip zero-index metamaterials integrated with silicon pillar array at *λ* = 1,570 nm [14].

On the other hand, the directive emission has been demonstrated using a structural coherence of emitters. In the presence of a structural coherence of emitters in a sub-wavelength scale, a directive emission occurs owing to the phase coherence of the emitted EM waves. One simple example is the plasmonic Yagi-Uda antennas for high collection efficiency in angle-resolved cathodoluminescence spectroscopy [15]. We note that spatial coherence coming from null phase advance allows wavefront engineering through the geometric shape of the ENZ medium, while the structural coherence enables beam steering based on phase interference of the emitters.

Furthermore, despite efforts to achieve an efficient emission directionality using sub-wavelength structures of metallic plasmonics or inorganic dielectrics, barriers remain for biological sensing and imaging applications due to the lack of biocompatibility and the complexity of sample fabrication. In this work, we explore directive emission by using one of the polymeric fluorophores, regioregular poly(3-hexylthiophene-2,5-diyl) (rr-P3HT) [16], in the presence of organic ENZ film of polymethine dye, (squaraine, HTJSq) [17–20]. The rr-P3HT fluorophore is chosen due to two key reasons, first is the emission spectral range overlap with the ENZ region of HTJSq from 600–690 nm, and second is the tendency to aggregate at higher concentrations [21]. Thus, through aggregation the structural coherence can be manipulated in the rr-P3HT film and a spatial coherence from the ENZ HTJSq film is predictable. Although there are other fluorophores that have higher PLQY, investigating the changes in directionality and emission characteristics was the primary objective where the detectable PL intensity of rr-P3HT was adequate to perform the investigation. Results show that a 42 % increase in directive emission efficiency is achieved once a structural coherence in regioregular fluorescent polymeric film and ENZ dispersion in organic squaraine film are introduced simultaneously.

## 2 Results and discussion

### 2.1 Sample preparation and dielectric spectrum

The rr-P3HT films are prepared by spin-coating from two different concentrations of P3HT:chlorobenzene (CB) solutions (rr-P3HT:CB = 60 mg/mL and 120 mg/mL) as described elsewhere [22, 23]. Figure S1a in Supporting Information shows the chemical structure of rr-P3HT. In previous studies, the high-concentration of 120 mg/mL rr-P3HT (rr-P3HT-120), 560 nm thick, films showed a higher molecular alignment and a high degree of crystallinity as compared to the low-concentration of 60 mg/mL rr-P3HT (rr-P3HT-60), thickness of 320 nm, films [22]. The concentration-dependent crystallinity of rr-P3HT films is shown through the grazing incidence X-ray diffractograms in Figure S1b, and the emission spectra in Figure S1c, the absorption spectra in Figure S1d and PL lifetime spectra in Figure S1e. The spectral peak shift in both emission and absorption spectra of higher concentration rr-P3HT film indicates the high degree of crystallinity resulting from molecular aggregation. Owing to the concentration-dependent interchain interactions of the rr-P3HT molecules, a higher structural coherence of the transition dipole is expected in the high-concentration rr-P3HT-120 film than in the low-concentration rr-P3HT-60 film [16].

**Figure 1:**
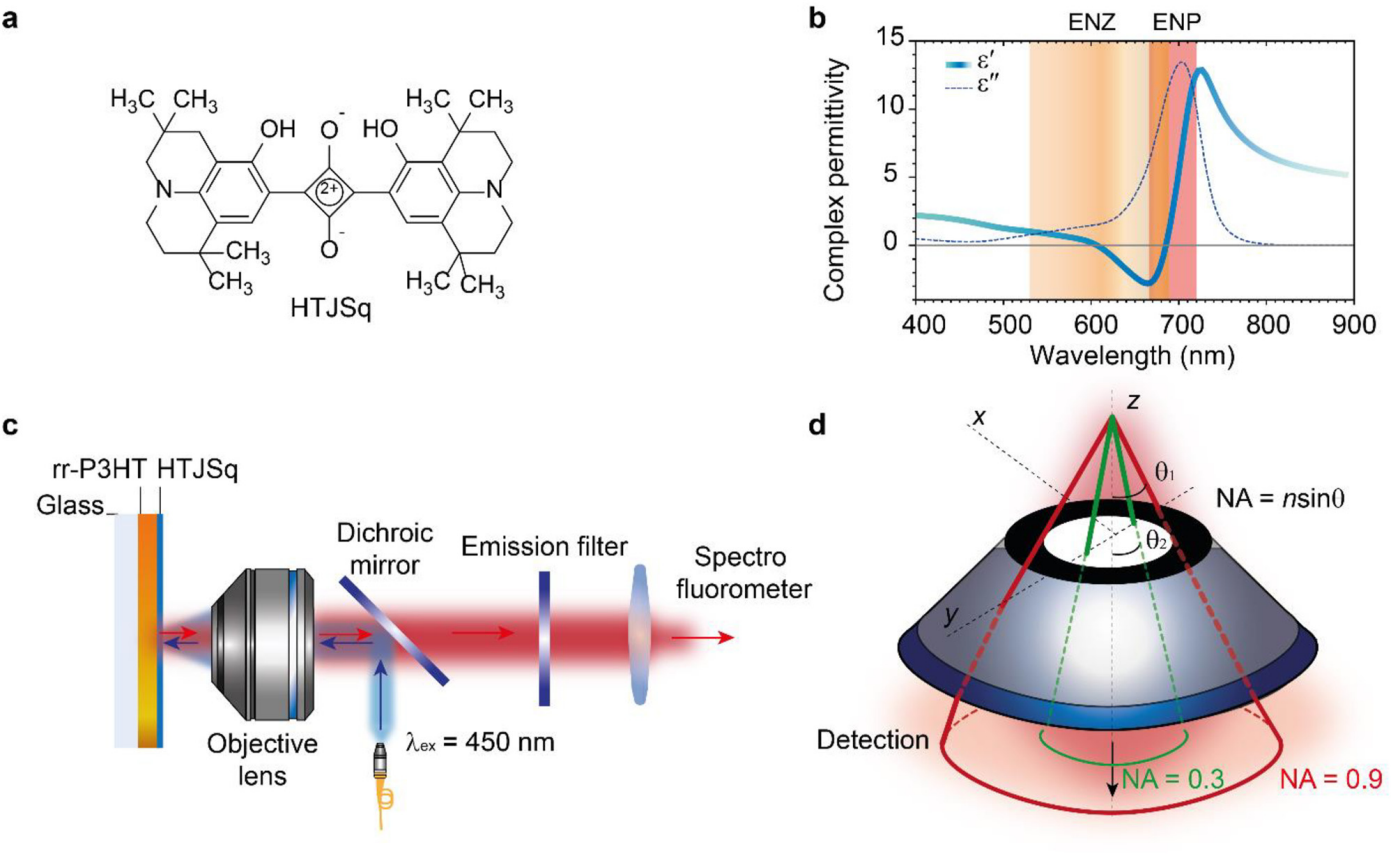
Schematic of (a) chemical structure of HTJSq molecule is displayed along with (b) complex permittivity spectra of 5 nm thick HTJSq film. (c) Optical experiment setup for steady-state photoluminescence microscope measurement. An excitation laser (*λ* = 450 nm) and objective lens are used to measure the emission intensity of the rr-P3HT film. (d) Schematic of directive emission measurement with microscope objectives with numerical aperture NA = 0.3, in green, and NA = 0.9, in red.

In order to identify the presence of an ENZ spectral range in HTJSq film, HTJSq squaraine [17] (see Figure 1(a) for molecular structure) is thermally evaporated onto fused silica substrates to obtain 5 nm-thick HTJSq film. Dielectric spectra of the film is obtained from spectroscopic ellipsometry measurement (Supporting Information S2), plotted in Figure 1(b). The HTJSq film exhibits an ENZ response in the spectral range of 530–690 nm, shown in light orange, with an epsilon-near-pole (ENP) response in the spectral range of 690–725 nm, shown in dark orange [17]. While the Drude model explains the response from non-bound free electrons, particularly in metals, the Lorentz model fits to a description of bound electron response. In polymethine molecular family to which HTJSq belongs, a strong donor strength in donor–acceptor–donor architecture is associated with a narrow full-width-half-maximum of both absorption and emission spectra as well as a nearly vanishing Stokes’ shift. In particular, the narrow strong absorption, i.e., a highly absorptive dielectric permittivity, correlates with a highly dispersive dielectric permittivity via Kramers–Kronig relation [20]. The characteristic Lorentzian dispersion of polymethine dye allows for organic ENZ materials to obtain an ENZ response for a particular spectrum range, here for the visible spectrum range of 530–690 nm.

### 2.2 PL spectra measurement for directive emission

**Figure 2:**
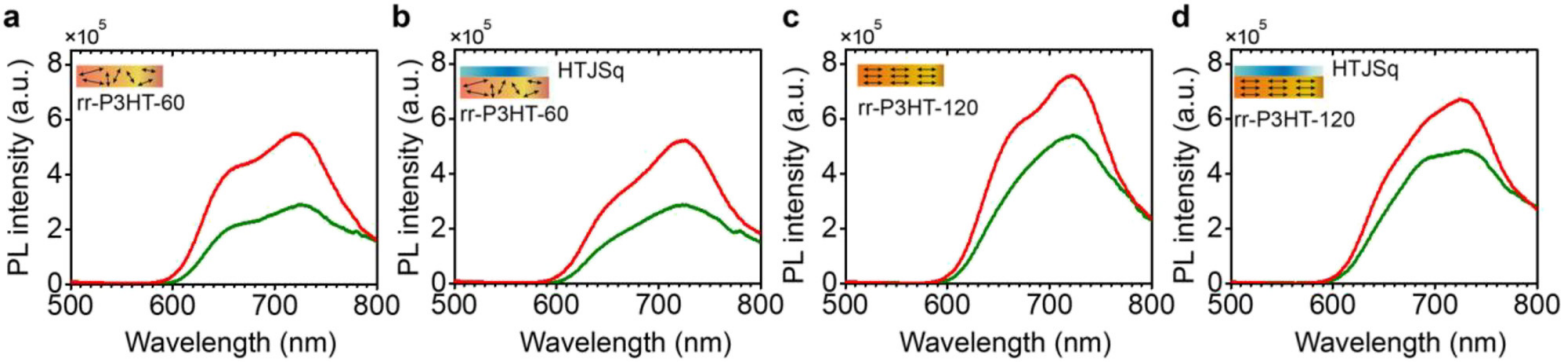
PL spectra of rr-P3HT-60 film (a) bare, (b) with HTJSq film on top, and PL spectra of rr-P3HT-120 film (c) bare, and (d) with HTJSq film on top collected by NA 0.3 (green curve) and NA 0.9 (red curve) microscope objectives are displayed.

Next, in order to examine the directive emission in the presence of the HTJSq ENZ film, the HTJSq molecular layer is thermally evaporated on top of rr-P3HT films of 60 mg/mL and 120 mg/mL concentrated films. Steady-state PL microscope with reflection geometry measurements were performed using microscope objectives with numerical aperture (NA) of 0.3 (10×) and 0.9 (60×), as shown in Figure 1(c) and (d). The excitation laser of 450 nm is reflected off a 488 nm dichroic mirror toward specimen and then emission spectrum from the rr-P3HT film collected by a 488 nm long pass filter. The degree of directive emission is determined by measuring the PL intensity with the two objectives and calculating the ratio of the measured intensities. For example, if the PL intensities are approximately equal for the two separate measurements with low and high NA objectives, i.e., if the ratio of measured intensities with low and high are about one, most of the collected PL intensity originates from the central part subtended by a very small angle *θ* in Figure 1(d). That is, a higher (lower) ratio of 0.3 versus 0.9 NA corresponds to a higher (lower) directionality of the PL emission [24]. The PL spectra of bare rr-P3HT-60 film collected with a NA 0.3 (green curve) and NA 0.9 (red curve) microscope objectives are plotted in Figure 2(a). The PL spectra of rr-P3HT-60 film with HTJSq film on top is shown in Figure 2(b). In the same manner, PL spectra of bare rr-P3HT-120 film are plotted in Figure 2(c) with the PL spectra of rr-P3HT-120 film with HTJSq on top shown in Figure 2(d). Now, the directive emission is examined by comparing the ratio of measured PL intensities using the two objectives. PL intensity integrated over the entire PL spectral range for each microscope objective is listed in Table 1, along with the ratio of NA 0.3 versus NA 0.9 microscope objective collections.

**Table 1:** Integrated steady-state PL intensities of Figure 2(a–d).

	rr-P3HT-60				rr-P3HT-120			
			0.3 NA	0.9 NA	0.3 NA versus 0.9 NA		0.3 NA	0.9 NA	0.3 NA versus 0.9 NA
Bare	(a)	3.9	7.1	0.55	(c)	6.9	9.4	0.73
HTJSq	(b)	3.7	6.4	0.58	(d)	6.6	8.5	0.78

Before proceeding to examine directive emission resulting from the presence of HTJSq ENZ film, we compare the PL emissions from rr-P3HT-60 and rr-P3HT-120 films in Figure 2(a) and (c), with integrated PL intensity ratio listed in the 1st row of Table 1. Interestingly, the PL emission is more directive in the bare rr-P3HT-120 with a ratio of 0.73 compared to the bare rr-P3HT-60 with a ratio of 0.55. In addition, a comparison of the NA 0.9 collections (red curves) of Figure 2(a) and (c) shows that both PL peaks at ≈ 655 nm and ≈ 730 nm exhibit directive emission, maintaining the overall PL spectral shape. This is attributed to an increased in-plane horizontal dipolar emission in the rr-P3HT-120 with the structurally coherent alignment of rr-P3HT molecules and high crystallinity [21].

Now we closely examine how the presence of the HTJSq ENZ film enhances the directionality of PL from the rr-P3HT film. From the comparison of intensity ratios of 0.55 for bare rr-P3HT-60 of Figure 2(a) and 0.58 for rr-P3HT-60 with HTJSq ENZ film on top of Figure 2(b), it is evident that the presence of HTJSq ENZ film enhances the directionality of PL emission from rr-P3HT-60 film. There is a substantial overlap of the blue part of the rr-P3HT film PL spectrum with the ENZ spectral region of the HTJSq film, 530–690 nm. The spatial coherence established in an optical EM wave inside ENZ medium leads to a directive emission upon exiting the HTJSq ENZ film located on top of polymeric fluorophore rr-P3HT film.

For the case of rr-P3HT-120, PL intensity ratios of 0.73 for bare rr-P3HT-120 of Figure 2(c) and 0.78 for rr-P3HT-120 with HTJSq ENZ film of Figure 2(d) also evidences that the presence of HTJSq ENZ film enhances the directionality of the PL emission. In addition, a high PL intensity ratio for the bare rr-P3HT-120 is from the fact that most of the transition dipoles in rr-P3HT-120 film are already oriented along the in-plane direction of the film, namely, the horizontal direction, exhibiting a directive emission resulting from structural coherence [22].

A sectional investigation on the spectral dependent PL intensity ratio is conducted in S3 (see Supporting Information). From Table S2 and S3 a clear enhancement of directionality is observed in the ENZ spectral range of HTJSq film, with PL intensity ratio increase of 0.48–0.53 for bare rr-P3HT-60 to with HTJSq ENZ film, and 0.64 to 0.72 for the case of rr-P3HT-120. However, for the spectral region where HTJSq film is dielectric, no PL ratio intensity change is observed with and without the HJTSq film. A constant PL intensity ratio is observed with value of 0.65 for both bare and with HTJSq film for rr-P3HT-60 and 0.83 for both bare and with HTJSq film with rr-P3HT-120.

**Figure 3:**
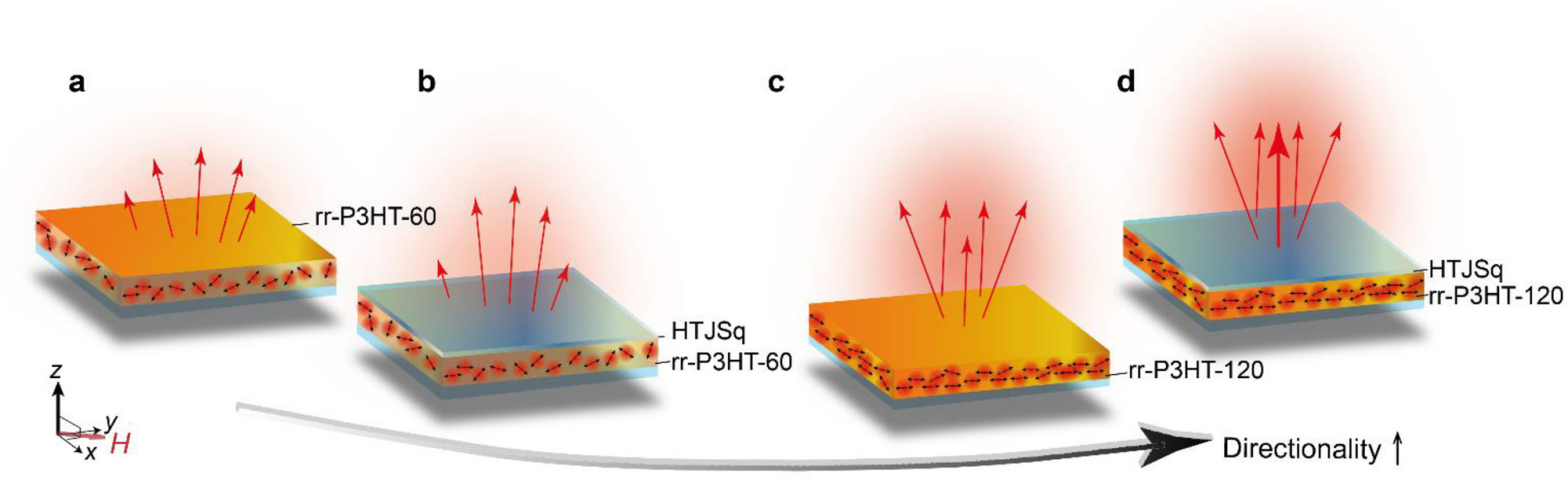
Schematics of directional emission from (a) rr-P3HT-60 bare film, (b) rr-P3HT-60 film with HTJSq film on top, (c) rr-P3HT-120 bare film, and (d) rr-P3HT-120 film with HTJSq film on top.

Figure 3(a) shows a schematic of PL emission from the bare rr-P3HT-60 film composed of incoherently structured exciton transition dipoles, while a spatial coherence from the HTJSq ENZ film located on top leads to a directive emission in Figure 3(b). In contrast, Figure 3(c) displays a schematic for the directive emission from a highly ordered bare rr-P3HT-120 film, namely, from a structural coherence of exciton transition dipoles. In Figure 3(d) a highly directive emission of PL from the rr-P3HT results from structural coherence of the exciton transition dipoles in the molecular structure of rr-P3HT-120 film as well as an enhanced spatial coherence of the optical EM wave associated with null phase advancement in the HTJSq ENZ film located on top.

### 2.3 Simulation

**Figure 4:**
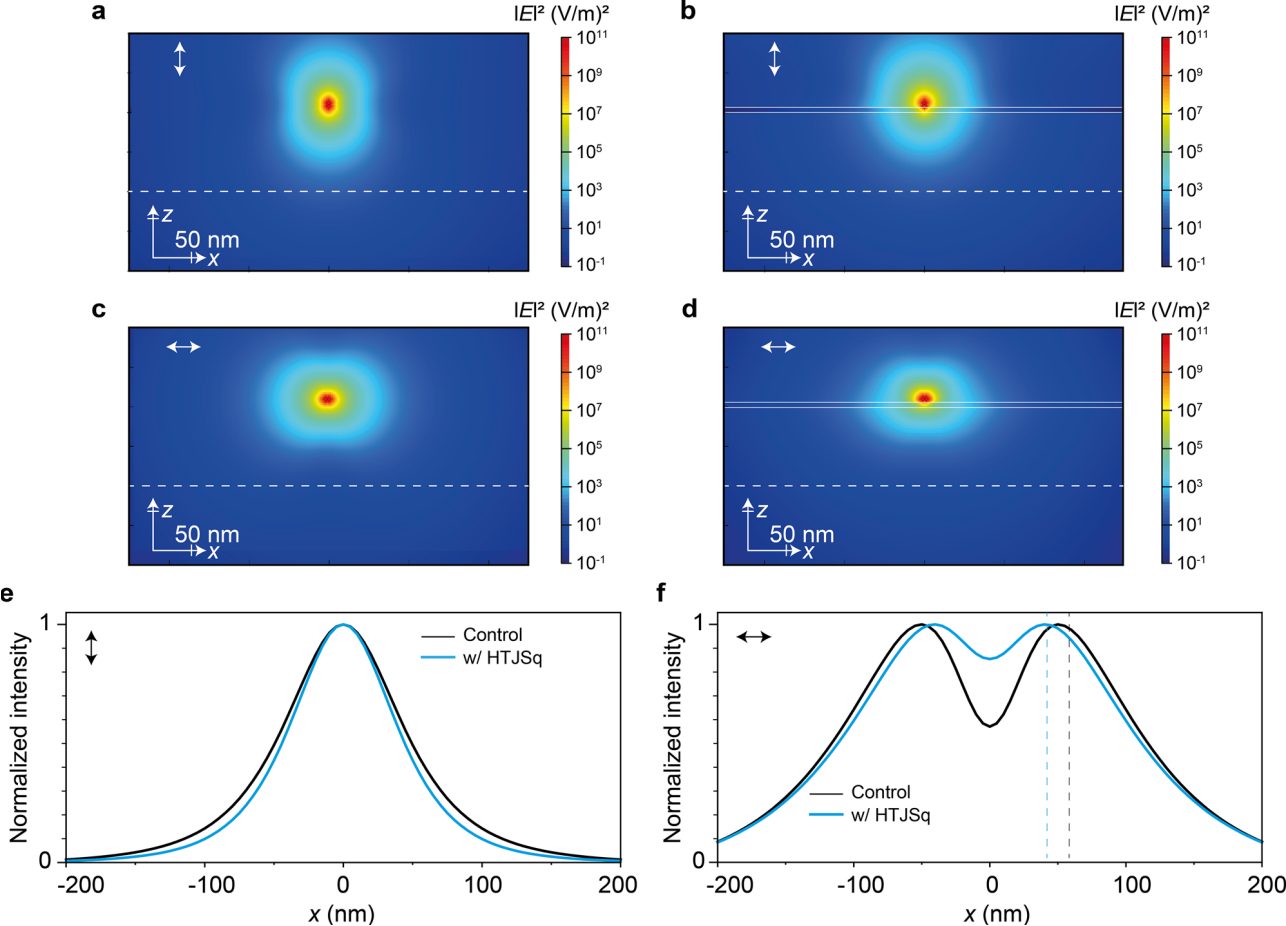
Electric field intensity distribution of a vertically (↕) oriented electric dipole source located (a) in vacuum, (b) near the 5 nm-thick HTJSq film. Electric field intensity distribution of a horizontally (↔) oriented electric dipole source located (c) in vacuum, and (d) near the 5 nm-thick HTJSq film. Spatial distribution of normalized intensity patterns of (e) vertically (↕) and (f) horizontally (↔) oriented electric dipole source. The emission intensity of the dipole was detected with a power monitor located 100 nm below the dipole source location in (a)–(f).

The emitted power of the electric dipole located near an ENZ medium has been investigated in literature [23]. We have calculated the electric field distribution in the presence of HTJSq film by using finite difference time domain (FDTD) simulation. Figure 4(a–d) show the intensity distribution emitted from a vertically (↕) or horizontally (↔) oriented electric dipole Figure 4(a and c) in vacuum, Figure 4(b and d) placed on top with HTJSq film, respectively. As can be seen in Figure 4(e and f), the normalized intensities, in the presence of HTJSq ENZ film (blue curve), show a narrower beam width compared with that in vacuum (black curve).

In rr-P3HT-120 film, the emission dipoles are orientated predominantly along the *x*–*y* horizontal plane, namely, oriented predominantly in horizontal (↔) direction. Relation of the ratio tendency 0.73(bare) <0.78 (HTJSq) in the right column of Table 1 is consistent with the simulated results of Figure 4(f), where the difference between HTJSq and bare film is found to be 0.03.

In rr-P3HT-60 film, the emission dipoles are almost randomly oriented along the *x*-, *y*- and *z*-axis [22]. In the left column of Table 1, the ratio of 0.55 (bare) <0.58 (HTJSq) is in line with the tendency of the simulated results of Figure 4(e) and (f), where the normalized intensities from both vertically (↕) and horizontally (↔) oriented electric dipoles show the beam widths narrowed.

**Figure 5:**
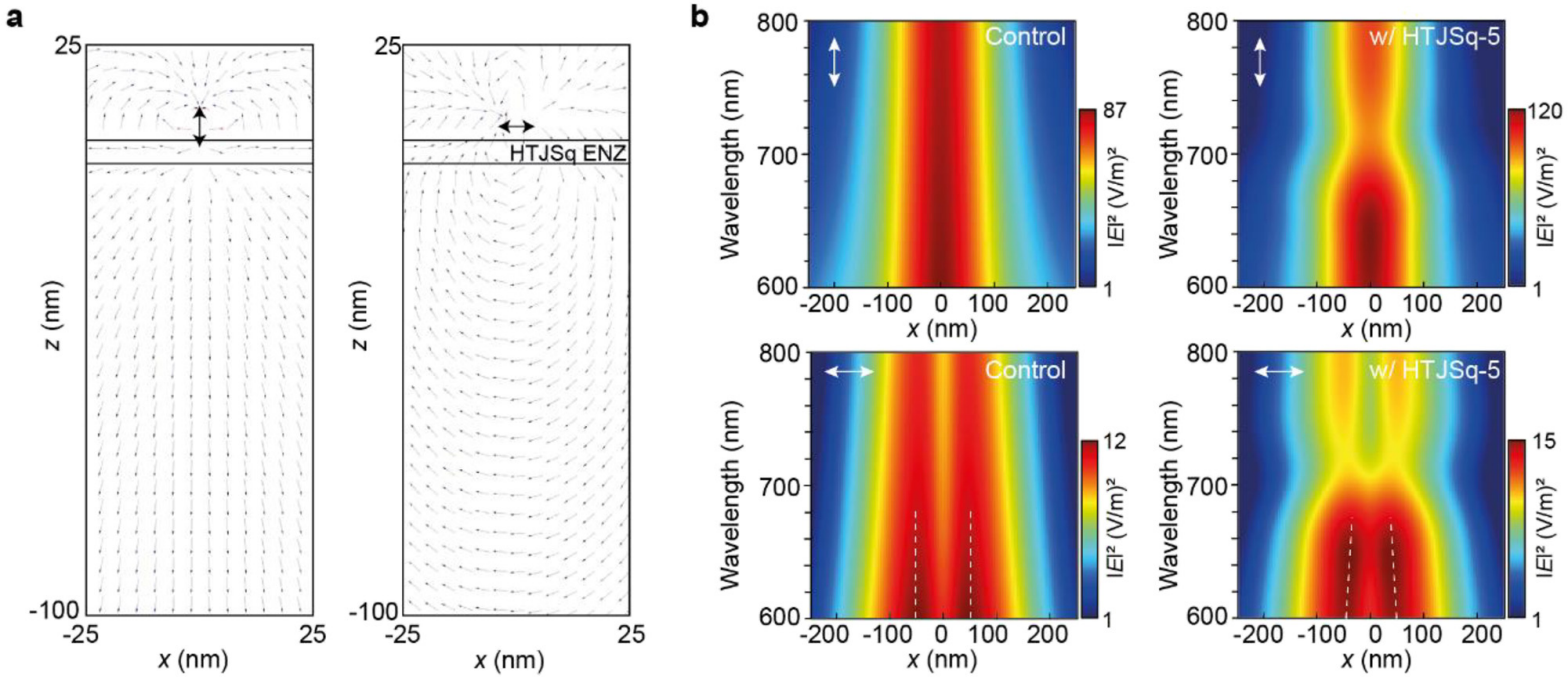
2D plot of (a) the simulated electric field vectors in the presence of the 5-nm thick HTJSq ENZ film for vertically (↕, left panel) and horizontally (↔, right panel) oriented electric dipole at *λ* = 685 nm and (b) the simulated electric field intensity.

Calculation of the electric field for vertically (↕, left panel) and horizontally (↔, right panel) oriented electric dipole placed on top of the HTJSq ENZ films was carried out by FDTD. In Figure 5(a), the directions of transmitted electric field vectors are more uniform compared to those in the absence of HTJSq ENZ film (not shown here). It can be seen that the electric field vectors are significantly redirected by the presence of the HTJSq ENZ film. Indeed, the electric field vectors of the dipole in Figure 5(a) shows that the presence of HTJSq ENZ film behaves as an angular filter at *λ* = 685 nm, allowing transmission for only a specific narrow angular width close to the normal [11, 13]. Directional filtering of transmission allows for the transmitted electric field of the dipole to behave like a plane-wave distribution as it passes through the HTJSq ENZ film. We simulated transmitted intensity distributions from vertically (↕, Figure 5(b) upper panel) and horizontally (↔, Figure 5(b) lower panel) oriented electric dipoles located near the HTJSq ENZ film, with emission wavelengths from 600 to 800 nm. The transmitted intensity monitored at 100 nm below the position of the dipole is calculated as shown in Figure 5(b). The presence of an HTJSq ENZ film (right panel of Figure 5(b)) results in a pronounced narrowing of the intensity distributions in the ENZ spectral range as compared to the control sample (left panel of Figure 5(b)).

## 3 Conclusions

Organic epsilon-near-zero HTJSq molecular film is shown to enhance directionality of the photoluminescence emission from rr-P3HT polymeric fluorophore. Spatial coherence of optical wave originating from the ENZ response in squaraine film is responsible for the directive emission of photoluminescence. Notably, HTJSq ENZ film offers wavelength tunability for controlling the directive emission at optical frequency. Based on purely organic compositions, the emission directionality is increased by 42 % at visible wavelength spectral range by taking advantage of the spatial coherence in squaraine film and structural coherence in polymeric fluorophore film. Organic ENZ film is highly useful for realizing directive emissions in applications such as sensing and imaging where bio-compatibility of fluorophore-embedding medium is required.

## 4 Methods

### 4.1 Sample preparation

Regioregular Poly(3-hexylthiophene-2,5-diyl) (rr-P3HT) with molecular weight 42,000 is purchased from Sigma-Aldrich, which is dissolved in chlorobenzene (CB) in concentrations of 60 mg/mL and 120 mg/mL. Solutions are subsequently heated at 70° for 3 h using a hotplate to ensure total dissolution. Films are fabricated by spin-coating onto fused silica substrates at 5000 rpm for 60s. Solutions of 60 mg/mL and 120 mg/mL provided 250 nm- and 800 nm-thick P3HT films, respectively. Substrates were forehand washed in acetone, isopropyl alcohol (IPA), and chloroform (CHCl_3_) accordingly for 10 min each in an ultrasonic bath, with excess liquid blown off with N_2_ gas. HTJSq molecule is thermally evaporated for ENZ film onto the rr-P3HT films with the evaporation rate of 0.20 ∼ 0.45 ^Å^/s in a vacuum chamber of 7.9 × 10^−6^ Torr.

### 4.2 SSPL/SSPL microscope and directive emission measurement

Steady-state photoluminescence (SSPL) is performed on rr-P3HT and rr-P3HT-HTJSq films in both direct point PL and microscope PL, using a Horiba Jobin Yvon fluorimeter (FluoroMax-4). Microscope objectives (Olympus) with numerical apertures 0.3 and 0.9 are used, which are chromatically corrected for red and blue with correction level of chromatic aberration being semiapchromat (FL).

### 4.3 Spectroscopic ellipsometry

The real and imaginary parts of the dielectric constants were determined through spectroscopic ellipsometry (SE) using Woollam co. alpha-SE spectroscopy ellipsometer with measurement range of 381 nm–887 nm and single incidence angle of 70°. Both parameters of Ψ and ∆ are measured corresponding to amplitude tan(Ψ) and phase e^*i*∆^ of the reflected elliptical polarization state after incident linearly polarized light with light beam of diameter of ∼2 mm. See Supporting Information for more details.

### 4.4 Finite-difference time-domain simulation

Finite-difference time-domain (FDTD) simulations were used to calculate the electric field distribution. Oscillating dipoles are used to simulate point sources such as fluorescent molecules. The simulation volume is a 1 × 1 × 1 µm^3^ with the 3D uniform grids, and a minimum mesh size is 0.25 nm. Non-physical reflections on the simulation walls are suppressed by perfectly matched layer (PML) boundary conditions along the *x*-, *y*- and *z*-axis. Once the 3D uniform mesh grids have been created, electric field vectors can be extracted in the Lumerical FDTD scripting window or in MATLAB. Emitted intensity distribution of the dipole source and a vector plot of the electric field distribution were calculated by a *xz*-plane power monitor. A 1D plot of the dipole’s emission intensity distribution was extracted from a 1D *x*-power monitor placed at *z* = 100 nm and *y* = 0 nm. A nested parameter sweep method was used to generate a 3D plot of the intensity distribution as a function of position *x* and the center wavelength of the emitted dipole. The start/stop values of the wavelengths to sweep were set from 600 nm to 800 nm.

## Supplementary Material

Supplementary Material Details
