# Supplementary material for: Directive emission from polymeric fluorophore with epsilon-near-zero squaraine molecular film
Source: Nanophotonics. 2023 Apr 25;12(13):2471–8. doi: 10.1515/nanoph-2022-0763 (PMC11501559; doi:10.1515/nanoph-2022-0763)
Supplement: Supplementary file 1 — Supplementary Material Details [file j_nanoph-2022-0763_suppl.pdf]

Directive emission from polymeric fluorophore with epsilon-near-zero squaraine molecular film

Kyu-Ri Choi[†], Minjae Kim[‡], Jeong Weon Wu[‡,*], Anthony D'Aléo[§,*], and Yeon Ui Lee[†,*]

† Department of Physics, Chungbuk National University, Cheongju, Chungbuk, 28644, South Korea

‡ Department of Physics, Ewha Womans University, Seoul, 03760, South Korea

§ Université de Strasbourg, CNRS, Institut de Physique et Chimie des Matériaux de Strasbourg, UMR 7504, F-67000 Strasbourg, France

yeonuilee@cbnu.ac.kr, anthony.daleo@ipcms.unistra.fr, jwwu@ewha.ac.kr

Supporting Information

S1. Characterization of rr-P3HT films

S2. Spectroscopic ellipsometry characterization of HTJSq film

S3. Sectional integrated steady-state photoluminescence (SSPL) intensities

S1. Characterization of rr-P3HT films

To produce distinct crystalline structures in the emissive layer, 60 and 120 mg portions of regioregular poly(3-hexylthiophene-2,5-diyl) (rr-P3HT; **Fig. S1a**) were each dissolved in 1 mL portions of chlorobenzene. The resulting solutions were heated at 70˚C for 3 h (rr-P3HT:CB = 60, 120 mg/mL) and spin-coated onto fused silica substrates as described in Ref.[1]. **Fig. S1b** shows the grazing incidence X-ray diffractograms (GIXD) obtained with a simple X-ray diffractometer for the self-assembled rr-P3HT films with different concentrations (60 and 120 mg/mL). It is well known that the differences in the degree of crystalline order affect the emission spectra.[1-3] Compared with that of low concentration rr-P3HT film (60 mg/ml) of 320nm thickness (**Fig. S1c**, blue curve), the high concentration rr-P3HT film (120 mg/ml)

of 560nm thickness (**Fig. S1c**, red curve) shows red-shifted emission peaks. The intensity of the low energy peak (721 nm) is notably higher than the high energy peak (~650 nm), indicating a high degree of crystallinity resulting from molecular aggregations.[1-3] The aggregation characteristics were further confirmed by measuring absorbance spectra and a blue/red-shift of absorption peak indicates H/J-molecular aggregation (**Fig. S1d**). **Fig. S1e** shows a shortening of photoluminescence (PL) lifetime resulting from an increased radiative decay rate in a highly crystalline aggregates of high-concentration film. The high concentration rr-P3HT film (120 mg/mL) has a highly ordered uniaxial lamellar crystalline structure (**Fig. S1**)[1]; therefore, the in-plane transition dipole moments of the high concentration rr-P3HT film lead to a structural coherence in emission.

1. Lee, Y. U., Yim, K., Bopp, S. E., Zhao, J. & Liu, Z. Low-Loss Organic Hyperbolic Materials in the Visible Spectral Range: A Joint Experimental and First-Principles Study. *Adv. Mater.* **32**, 2002387 (2020).
2. Scharsich, C. *et al.* Revealing structure formation in PCPDTBT by optical spectroscopy. *J. Polym. Sci. Part B Polym. Phys.* **53**, 1416–1430 (2015).
3. Eder, T. *et al.* Switching between H- and J-type electronic coupling in single conjugated polymer aggregates. *Nat. Commun.* **8**, 1641 (2017).

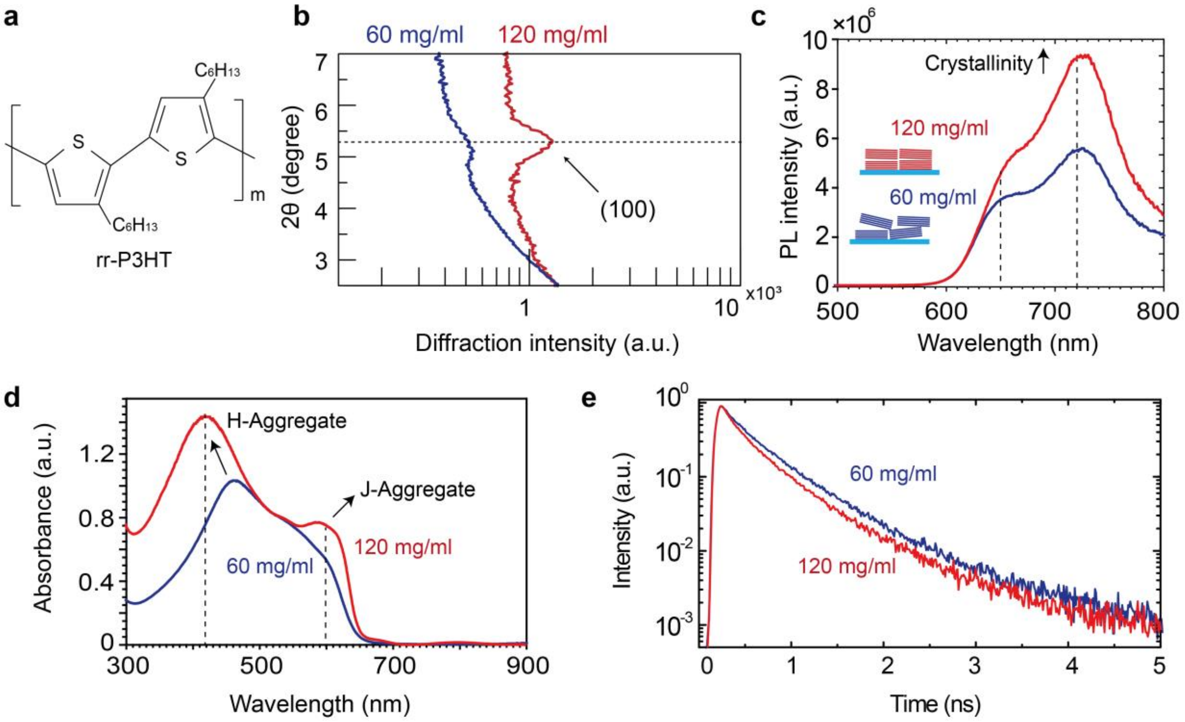

Figure S1. (a) Structure of rr-P3HT polymer, (b) grazing incidence X-ray diffractograms, (c) emission spectra, (d) absorbance spectra, and (e) PL lifetime of rr-P3HT films with different concentrations (60 and 120 mg/mL).

S2. Spectroscopic ellipsometry Characterization of HTJSq film

Spectroscopic ellipsometry measurements are conducted on a thermally evaporated HTJSq film in the spectral region of 380-980 nm. The ratio between reflected *p*- and *s*-polarized light are measured at different incidence, from which the amplitude ratio (tan Ψ) and phase difference (Δ) is obtained. The measured (back and green curves) and fitted (red dotted line) Ψ and Δ of HTJSq film, shown in **Fig. S2** for an incidence angle of 70°. To obtain the complex permittivity of HTJSq film, an air-thin film-substrate model provided by CompleteEASE® data analysis software was used. The Fresnel reflection coefficients were calculated by using an iterative non-linear regression algorithm (the Levenberg-Marquardt method). The dielectric permittivities were simulated through the sum of Gaussian oscillators with details listed in Table S1 for HTJSq film. 6 Gaussian oscillators were used for HTJSq film.

In addition, in order to identify the optical isotropy of a thermally evaporated HTJSq film, Ψ and Δ were measured for HTJSq film with incidence angles of 65°, 70°, and 75°, as shown in **Fig. S3**. The results show that the extracted permittivity values are consistent for various incident angle measurements, with a reasonable estimate of assumption that the thermally evaporated HTJSq film is optically isotropic.

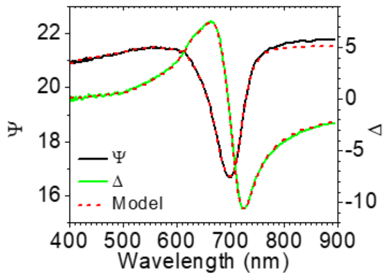

Figure S2. Ψ and Δ for incident angle of 70° HTJSq film

Table S1. Oscillator list for dielectric permittivity of thermally evaporated HTJSq film

NUMBER	TYPE	AMPLITUDE	BROADENING	CENTER ENERGY
**1**	Gaussian	0.906	0.535	2.241
**2**	Gaussian	1.488	0.133	1.915
**3**	Gaussian	7.961	0.150	1.780
**4**	Gaussian	2.333	0.072	1.741
**5**	Gaussian	1.354	0.364	1.937
**6**	Gaussian	0.265	0.658	3.055

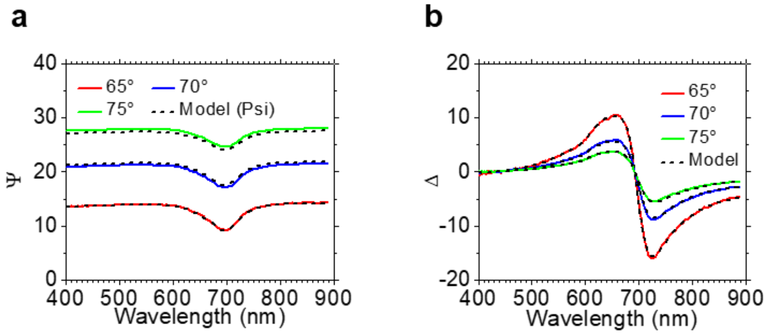

Figure S3. Ψ and Δ for incident angles of 65°, 70° and 75° of HTJSq film

S3. Sectional integrated steady-state photoluminescence (SSPL) intensities

A sectional integration of PL was measured to compare the effect of the HTJSq ENZ film. The observed ENZ region of 600-690 nm was selected by the overlapping HTJSq ENZ spectral region with the P3HT emission range, Fig. S4 (cyan). The dielectric region was sectioned from 725-800 nm, Fig. S4 (orange). The PL intensity integration for each section is shown in the colour-coordinated tables of Table S2 and Table S3. As can be seen from Table S2, in the ENZ region of 600-690 nm, the PL emissions are more directive with the HTJSq film than without. Thus, the intensity ratio in the case of rr-P3HT-60 increased from 0.48 to 0.53 and rr-P3HT-120 increased from 0.64 to 0.72.

On the other hand, it is interesting to note that in the region where HTJSq is dielectric, 725-800 nm, no ratio difference is observed showing no change in directionality. As shown in Table S3, the integrated PL intensity ratio in the case of both bare film or with the HTJSq film has the same ratio of 0.65 for rr-P3HT-60. For the case of rr-P3HT-120 both bare and with the HTJSq film gave the integrated PL intensity ratio of 0.83, indicating no directionality enhancement when the HTJSq film is dielectric.

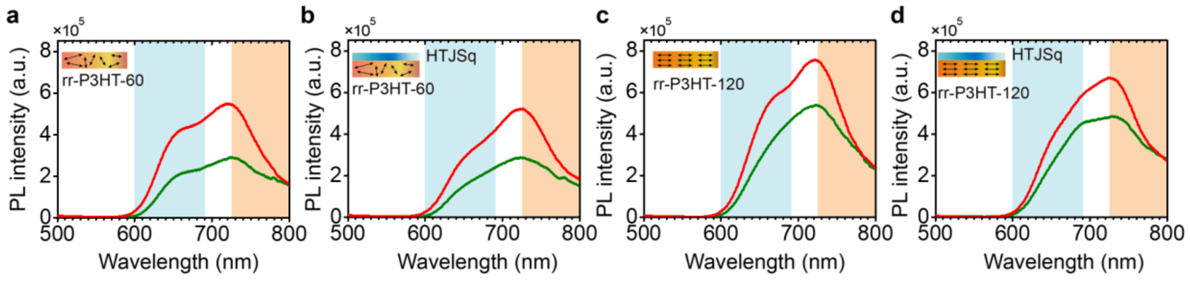

Figure S4. Section divided PL spectra of Fig. 2 with highlighted sections of HTJSq ENZ spectral region 600-690 nm (cyan), and dielectric 725-800 nm (orange). PL spectra of rr-P3HT-60 film (a) bare, (b) with HTJSq film on top, and PL spectra of rr-P3HT-120 film (c) bare, (d) with HTJSq film on top collected by NA 0.3 (green curve) and NA 0.9 (red curve) microscope objectives.

Table S2. Integrated SSPL intensities of Fig. 2 in the ENZ spectral region of 600-690 nm.

ENZ 600 - 690 nm	rr-P3HT-60					rr-P3HT-120		
		0.3NA	0.9NA	0.3NA *vs.* 0.9NA		0.3NA	0.9NA	0.3NA *vs.* 0.9NA
Bare	(a)	1.3	2.8	0.48	(c)	2.1	3.3	0.64
HTJSq	(b)	1.1	2.1	0.53	(d)	2.0	2.8	0.72

Table S3. Integrated SSPL intensities of Fig. 2 in the dielectric spectral region of 725 - 800 nm.

Dielectric 725 - 800 nm	rr-P3HT-60					rr-P3HT-120		
		0.3NA	0.9NA	0.3NA *vs.* 0.9NA		0.3NA	0.9NA	0.3NA *vs.* 0.9NA
Bare	(a)	1.7	2.6	0.65	(c)	2.9	3.5	0.83
HTJSq	(b)	1.7	2.6	0.65	(d)	2.9	3.5	0.83
